# Safety and Immunogenicity of a Human Papillomavirus Peptide Vaccine (CIGB-228) in Women with High-Grade Cervical Intraepithelial Neoplasia: First-in-Human, Proof-of-Concept Trial

**DOI:** 10.5402/2011/292951

**Published:** 2011-03-24

**Authors:** Ana M. Solares, Idania Baladron, Thelvia Ramos, Carmen Valenzuela, Zaida Borbon, Sonia Fanjull, Leonardo Gonzalez, Dagnelia Castillo, Julio Esmir, Milaid Granadillo, Aileen Batte, Alberto Cintado, Mayte Ale, Maria E. Fernandez de Cossio, Annia Ferrer, Isis Torrens, Pedro Lopez-Saura

**Affiliations:** ^1^Gyneco-obstetric Hospital Ramon Gonzalez Coro, Havana 10400, Cuba; ^2^Clinical Trials Division, Center for Biological Research, Havana 6996, Cuba; ^3^Gyneco-obstetric Hospital Mariana Grajales, Santa Clara 50100, Cuba; ^4^General Hospital Carlos Manuel de Céspedes, Bayamo 85100, Cuba; ^5^Department of Cancer, Center for Genetic Engineering and Biotechnology, P.O. Box 6162, Havana 10600, Cuba; ^6^Department of Genomics, Center for Genetic Engineering and Biotechnology, Havana 10600, Cuba

## Abstract

*Objective*. CIGB-228 is a novel therapeutic vaccine consisting of HLA-restricted HPV16 E7 epitope adjuvated with VSSP. This trial was designed to evaluate the toxicity, safety, immunogenicity, HPV clearance, and lesion regression. *Methods*. Seven women were entered. All were HLA-A2 positive, had biopsy-proven high-grade CIN, histologically positive for HPV16, and beared persistent postbiopsy lesions visible by digital colposcopy. HLA-A2 women with biopsy-proven high-grade CIN, HPV16-positive, and beared persistent postbiopsy lesions visible by digital colposcopy were vaccinated. One weekly injections of CIGB-228 vaccine was given for four weeks. Then, loop electrosurgical excision procedure (LEEP) of the transformation zone was performed. Study subjects were followed for 1 year after LEEP. *Results*. No toxicity beyond grade 1 was observed during and after the four vaccinations. Five of seven women had complete and partial regression. Cellular immune response was seen in all patients. HPV was cleared in three of the patients with complete response. *Conclusion*. CIGB-228 vaccination was well tolerated and capable to induce IFN*γ*-associated T-cell response in women with high-grade CIN. In several patients, lesion regression and HPV clearance were observed.

## 1. Introduction

The human papillomaviruses (HPVs), especially the high-risk types (HPV types 16 and 18) have been implicated in the development of high-grade cervical intraepithelial neoplasia (CIN II/III) and invasive cervical cancer [1, 2]. 

Preventive strategies utilizing vaccines directed against structural components of this virus have been approved. While prevention of HPV infection is an important goal for future generations of women, such intervention will useless to the millions already infected with high risk genotypes. 

Low-grade lesions (CIN I) usually clear spontaneously, whereas high-grade CIN II and III often persist and may progress to carcinoma [3, 4]. Current treatment strategies such as a loop electrosurgical excision procedure (LEEP) or cone biopsy of the cervix are aimed to excision of visible lesions and may eliminate HPV-infected cells and associated disease. Such ablative procedures may activate cellular immune responses to the associated HPV antigens. However, when the size of the lesion is large treatment with LEEP may be incomplete which leads to the permanence of positive margins and the persistence of high-grade lesions. Moreover, patients with CIN who have undergone ablative surgery or even a hysterectomy may still have HPV infection [5], which may result in recurrent or persistent CIN. These patients require repetitive invasive procedures for treatment. This can be problematic for women who desire future fertility due to distortion of the cervix after invasive interventions and also for preterm birth [6]. 

Vaccination strategies that boost natural immunity to HPV might allow clearance of the virus leading to resolution of dysplastic lesions. This could avoid the need for invasive treatment and may lead to a lower risk of recurrence. Various vaccines aimed at inducing regression in women with biopsy proven CIN II/III have been studied. The most frequently targeted antigens are the E6 and E7 proteins, because they are oncogenic and sustained expression is required for the maintenance of the cancerous phenotype. Their immunogenicity has rendered these HPV proteins an attractive target for immunization strategies to prevent cervical carcinoma [1, 2]. However, these vaccines have generally not been effective enough to produce a real tumor rejection (for review, see [7, 8]). As with every biological system, tumors use opportunistic and redundant mechanisms to guarantee their survival and development. The robustness of these processes makes cancer immunotherapy a real challenge. The success of cancer immunotherapy will not depend only on optimal tumor-associated antigen but also of the use of appropriate adjuvants, while being nontoxic and safe (for review, see [9]).

A new adjuvant approach has been previously developed in which gangliosides are incorporated into the *N. meningitides* outer membrane complex to form a nanoparticulate of very small size proteoliposomes (VSSP) [10]. Vaccination with the VSSP adjuvated HPV16 E7 (49–57) minimal CTL peptide protected mice against the HPV16 tumor model TC-1 challenge, induced regression of established tumors as well as E7-specific CD8^+^ T-cell responses [11].

On the basis of this preclinical rationale, “first-in-human” clinical trial with the CIGB-228 vaccine containing HLA-restricted HPV16 E7 epitope known to be recognized by CTLs (E7 peptide 86–93) [12] adjuvated with VSSP was done in patients with CIN II/III. In addition to the toxicity and tolerability of the CIGB-228 vaccine, immune, virological and clinical response end points were assessed.

## 2. Material and Methods

### 2.1. Patient Eligibility

HLA-A2-positive patients, 18-to-65 years-old with histological confirmed HPV16-positive CIN II/III, with a larger diameter ≥3 mm by videocolposcopy were recruited at specialized services from seven gyneco-obstetric hospitals throughout Havana and other provinces after oral and written informed consent (Table 1). They were then evaluated at the National Reference Center for Cervical Cancer at the “Ramon Gonzalez Coro” Havana Hospital, where the eligibility criteria were verified. Eligibility also required the following criteria: leukocytes >3 × 10^9^/L, lymphocytes >1 × 10^9^/L, thrombocytes >100 × 10^9^/L, and hematocrit >30%. Exclusion criteria were to have received any immunomodulator treatment up to 3 months before inclusion, psychiatric dysfunctions, pregnancy, and breastfeeding, decompensate chronic diseases such as asthma, epilepsy, autoimmune, or immunodeficiency diseases, hypertension, anemia, acute systemic or genital tract infections, and renal, hepatic, and cardiovascular disorders.

### 2.2. Study Design

**Figure 1:**
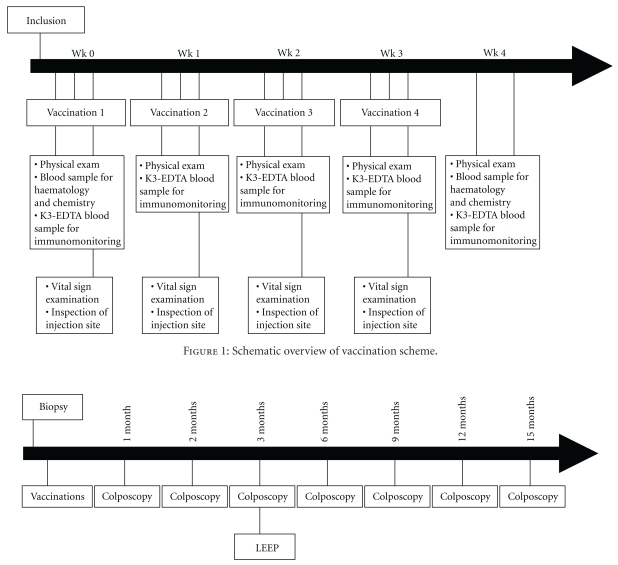
Schematic overview of vaccination scheme.

**Figure 2:**
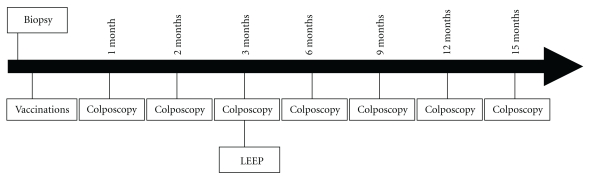
Schematic overview of the clinical trial procedure.

The study followed the principles of the Declaration of Helsinki for investigations in humans. It was approved by the Ethics and Scientific Committees of the participant institutions and by the Cuban Regulatory Authority. This was a single-arm, open, and uncontrolled study. The main purpose of the trial was to evaluate the product's safety during local and systemic adverse events. Sample size was previewed as between 7 and 10 patients. This N range assured that if no severe adverse reaction appeared, the probability of its occurrence would be less than 20%, with an 80%–90% confidence interval. Patients received four vaccinations. The vaccine was administered subcutaneously at 1-week intervals (Figure 1). Subjects were subjected to colposcopy monthly and LEEP excision of lesions in the cervix 60 days after completion of the immunization protocol (Figure 2).

### 2.3. Composition of the Vaccine

The CIGB-228 vaccine consists of two components. The HPV16 E7_(86–93)_ peptide was synthesized and supplied by the Center for Genetic Engineering and Biotechnology (CIGB) in 0.12 mg vials, as a lyophilized powder. VSSP was produced and supplied by the Center of Molecular Immunology in 0.8 mg/0.5 mL vials. Prior to the vaccination, the HPV16 E7_(86–93)_ synthetic peptide was reconstituted with water for injection and then adjuvated with VSSP. One dose of the CIGB-228 vaccine contained 0.1 mg of peptide and 0.16 mg of VSSP in a total volume of 0.5 mL.

### 2.4. Clinical Assessment

Subjects underwent a general physical, cytological, and a colposcopic examinations monthly and cervical biopsy prior to and at termination of the study by LEEP (90 days after the first vaccine administration). Blood samples were drawn for routine safety analysis prior to and 1 week after each vaccine administration and for immunological analysis prior to each vaccination and two weeks after the last dose (Figure 1). Patients were tested for pregnancy at each visit. Additionally, patients were followed clinically and colposcopically at 3, 6, 9, and 12 months after LEEP (Figure 2). Since the main purpose of the trial was to evaluate the product's safety, local and systemic adverse events were carefully assessed. Systemic toxicity was evaluated for 24 hours after each CIGB-228 vaccination, including continuous cardiovascular monitoring, temperature, respiratory frequency, and blood pressure measurements 30 min after each injection, then every hour during 4 hours. Patients completed diary cards of adverse events as auto report over the seven days following each vaccine administration. The medical terminology used for AE and their severity classification (in grades) were those of the Cancer Therapy Evaluation Program, Common Terminology Criteria [13]. 

Colposcopical evaluations were done using a video colposcopy device (Mediscope, Medison, Korea) with a calibrated rack to standardize imaging distance and illumination. Lesions were examined by two different specialists after applying 5% acetic acid for 3 min. They were described according to the Barcelona 2002 classification [14], and their morphometric analysis was done with validated software for quantitative digital image evaluation (MADIP V.4; Institute of Cybernetics, Mathematics, and Physics, Havana).

Histological analyses were performed on colposcopically directed biopsies from abnormal areas taken before treatment and afterwards in the LEEP specimen. Permanent sections were subsequently prepared, stained with hematoxylin-eosin, and finally reviewed by two different pathologists who classified the lesions according to the FIGO classification.

### 2.5. Response Criteria and Clinical Activity

The total lesion area (TLA) was the parameter evaluated for colposcopical response. Response was defined according to the RECIST criteria [15]. The percentage of decrease was measured as

(1) $$\text{\%decrease}=\frac{{\text{TLA}}_{\text{initial}}-{\text{TLA}}_{\text{final}}}{{\text{TLA}}_{\text{initial}}}\times 100.$$

Complete response was considered as disappearance of all initial TLA; partial response if at least a 30% decrease of the initial TLA; progressive disease meant at least a 20% increase of the initial TLA and as stable disease if a reduction or an increase of the TLA compared to the initial value that is not enough for classification of the outcome as partial response or progressive disease. 

The histological response was based on the lesions phase changes. A complete response was defined as the absence of high-grade lesions after vaccination, partial response when a downstaging of the high-grade lesion occurred; stable disease when a lesion remains at the same initial stage, and progression if there was upstaging.

### 2.6. HLA Testing

HLA-A2 allele was performed by PCR-SSP typing of HLA class I allele method [16].

### 2.7. Technique for HPV Detection

The presence of HPV DNA in biopsies was examined prior to and at termination of the study (90 days after the first vaccine administration) by PCR using a pair of L1-consensus primers (GP5+/GP6+) [17]. The HPV DNA detection limit was about 310 viral copies. Additional PCR with specific HPV16 primers was performed [18] and HPV DNA levels were normalized with those corresponding to *β*-globin as housekeeping gene.

### 2.8. Peptides

Peptides used for *in vitro* studies were synthesized at the CIGB. The peptide sequences were from HPV16 E7, encompassing amino acids 11–20, 82–90, and 86–93, and influenza M1 peptide (GILGFVFTL, aa58–66).

### 2.9. Lymphocytes

Peripheral blood mononuclear cells (PBMC) were isolated from fresh K3-EDTA blood samples within 2 h after sample collection using Ficoll density gradient according to the manufacturer (Bio-Sciences AB) and seeded at 5–10×10^6^ cells/mL in freezing medium consisting of nine parts fetal bovine serum (FCS; Hyclone,) and one part dimethylsulfoxide (Sigma). PBMC were stored overnight in 1°C freezing containers (Nalgene Nunc International) at −80°C and then transferred into liquid nitrogen until use.

### 2.10. HPV-Specific T-Cell Immunity Monitoring

Briefly, cryopreserved pre- and postvaccine PBMC were thawed quickly in a 37°C water bath. Then, cells were washed twice with phosphate buffer saline (PBS) and seeded in three replicate wells at a density of 2 × 10^5^ cell/well in 100 *μ*L of RPMI medium (Sigma) enriched with 10% of human AB serum (Sigma) and 50 *μ*L/well of 10 *μ*g/mL of indicated HPV16 E7 peptides, 10 *μ*g/mL of influenza M1 peptide and 0.1 *μ*g/mL of anti-CD3 mAb CD3-2 (positive control) or medium (negative control) as corresponded in a multiscreen 96-well PVDF plate (Mabtech AB) coated with an IFN-*γ*-catching antibody (Mabtech AB). After two days of incubation at 37°C, the ELISPOT was performed according to the manufacturer's instructions (Mabtech AB). Spots were counted with a fully automated computer-assisted video-imaging analysis system (Carl Zeiss Vision). A positive response to antigen was defined as >20 spots (IFN-*γ*-producing cells)/10^6^ PBMCs in response to antigen (after subtraction of background). A positive response after vaccination was recorded if the frequency of IFN-*γ*-producing cells postvaccination was greater than twice the prevaccination response to an antigen.

## 3. Results

### 3.1. Study Population

Between October 2007 and February 2008, 7 women were included. Clinical and demographic characteristics of the study patients are shown in Table 1. Age ranged from 24 to 43 years (median 29). All patients had high-grade CIN confirmed on their histological pre-study and lesion areas between 30 and 638 mm^2^ (Table 2). Moreover, all women were HPV16 and HLA-A2 positive. The treatment schedule was completed in all patients, which were also evaluated one year after their LEEP for recurrence.

### 3.2. Adverse Events

Table 3 lists all adverse events assessed as possibly being associated with the vaccine. All patients reported local pain at the vaccination site and 6 patients reported burning sensation. Other events were reported with low frequencies as local redness and swelling. Systemic adverse events, including fever, tremors, and cramps were registered in few cases. No vaccine-related events exceeded grade 1 (mild) and recovered spontaneously. One patient reported lower abdominal pain as a result of urinary sepsis. This event was considered to be unrelated to the vaccination. There were no late adverse events either.

### 3.3. Clinical Evaluations

Individual results are shown in Tables 1 and 2. TLA decreased in all patients at colposcopy at 3 months of followup after CIGB-228 vaccination. Colposcopic response was evidenced in six of seven patients (85.7%), four of them (57.1%) complete and two (28.6%) partial response (Table 2). Histological analyses indicated that 57.1% of the patients (4/7) experienced full regression while 14.3% (1/7) had histological grade downstaging. Stable disease was observed in two patients (28.6%). Concomitant negativization of HPV16 from the original lesion sites was observed in three of the patients who had a complete response (Table 1). None of the patients showed confirmed lesion recurrence during the 12-months, followup.

### 3.4. Immunologic Responses

**Figure 3:**
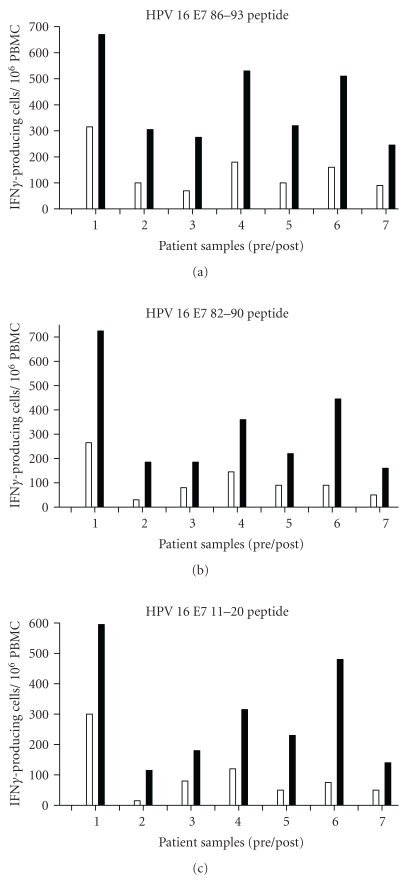
Immune response before and after vaccination. Note: ELISPOT data as number of spots per 10^6^ input CD8 cells is shown on the ordinate, with paired patient samples (prevaccine, white columns; postvaccine, black columns) indicated on the abscissa. Each panel is shown for the individual peptide indicated at the top. In each case, the maximum response postvaccination is shown.

PBMCs isolated from blood samples drawn weekly before each vaccination (first, second, third, fourth) and one week after the last vaccination were subjected to IFN*γ* ELISPOT analysis. Some preexisting HPV16 T cell immunity that was E7 peptides 11–20, 82–90 and 86–93-specific was detected in all patients (including fourth patients who had a complete response); this immunity was boosted by vaccination and coincided with enhanced IFN*γ* production for all patients. The immunologic analysis in which PBMCs were tested not only against the vaccination CTL peptide 86–93 but also two additionally E7 peptides (11–20, 82–90) not only measured the presence of specifically a vaccine-induced T-cell response but also allowed the calculation of the strength of the immune response, operationally defined as a combination of the magnitude and breadth of the T-cell response to all three peptide as intramolecular epitope spreading. After the vaccination, all seven patients responded to peptides 86–93 and 82–90. Six of seven patients were positive to peptide 11–20. The frequencies ranged between one HPV-specific T cell among 100,000 PBMCs up to 7 of 10,000 (see Table 4 for complete overview). The peak of response for each patient reached after two or three vaccinations, and the maximum response to HPV16 E7 86–93 was more vigorous than against HPV16 E7 82–90 and HPV16 E7 11–20 in the strength of response (Figure 3) and in general are correlated with the clinical outcome.

These results suggest that the clinical efficacy of a therapeutic HPV16 vaccine may be determined by its capacity to induce strong and broad immune responses to the HPV16 oncoprotein E7.

## 4. Discussion

The HPV is an attractive target for a vaccine strategy, since the HPV E7 transforming protein, which is critical for the maintenance of a transformed state in animal model systems and in humans, has been shown to encode epitopes that bind to class I alleles and are recognized by T cells. Virus-like particle vaccines have promise for primary prevention of cervical intraepithelial neoplasia and cancer; however, it is unlikely that they will impact on established disease or preexisting intraepithelial lesions, since the capsid proteins of HPV are not expressed by neoplastic cells in high-grade CIN. Women with HPV16 are more likely to develop CIN II/III than those with other HPV types, and these data provide a strong justification for devising immunization strategies against high-risk HPV type 16 to prevent progression of low-grade CIN to high-grade disease, to treat high-grade CIN, to prevent and treat the recurrence of high-grade CIN, and to prevent the occurrence of invasive cervical cancer. 

HPV-specific T cytolytic immune responses have been demonstrated in patients with high-grade CIN and cervical cancer [19]. Three peptides were defined that were immunogenic both in transgenic mice and in CTL induction experiments using PBMCs from HLA-A2 healthy donors derived from the 11–20, 82–90, and 86–93 amino acid sequences [20]. E7-specific CTL cells have been generated from the peripheral blood and lymph nodal tissue of HPV16 positive women with cervical dysplasia and cervical cancer by *in vitro* restimulation with autologous antigen-presenting cells pulsed with HPV16 E7 peptides [20, 21]. 

Trials to generate T cell reactivity against HPV16 E7 have been carried out in women with cervical cancer or cervical intraepithelial neoplasia. In previous reports, women with stage IV cervical cancer were immunized with an E7 86–93 lipopeptide sequence, and only six patients mounted a weak immune response against the E7 86–93 peptide sequence, without evidence of clinical benefit [22–24]. On the basis of the clinical rationale of that, women with a lower disease burden and preinvasive disease are more logical candidates for an antigen-specific immunotherapy than women with bulky invasive disease, prior chemotherapy, poor performance status, and profound immunosuppression; a phase I trial with a similar CTL peptide vaccine has been conducted in women with high-grade CIN. In that study, peptides binding to HLA-A2 emulsified with Montanide ISA 51, an oil-based adjuvant, were used for the treatment; 9/17 has evidence of clinical response with modest rates of immunity. Although the results obtained in that study were more optimistic respect to the clinical benefit, only 3 of 17 patients evaluated had complete regression of their lesion pathologically [25]. Due to the low levels of immunity seen and lack of regression observed, there is a perception that the peptide approach has little promise for control of HPV-induced cancer. That led many researchers to test vaccines based on E7 with other formats such as viral vectors, DNA vaccines, and fusion proteins [26–36]. Although there have been some encouraging results with these vaccines, so far, there is no clinical efficacy study in patients with cervical neoplasia and is still far to the approval of a therapeutic vaccine against HPV.

In this work, immunotherapy with CIGB-228 containing HPV16 E7 epitope adjuvated with VSSP is proven to be safe. The maximal toxicity seen was grade 2 and consisted of discomfort and swelling at the vaccination sites and low-grade fever in the first 24 h after injection. 

In the quoted studies, mostly, exact HLA class I-binding peptides were used for immunotherapy of metastatic melanoma, and the overall clinical results with such exact HLA-fitting peptides in patients with melanoma have been very disappointing worldwide [37]. However, our preclinical studies in mice indicated that immunization with exact MHC class-I-fitting peptide and VSSP is superior to that with peptide alone [11]. VSSP belong to the new generation of adjuvants based on pathogen-related molecules identified as “danger” signals that are recognized by the innate immune system [38]. VSSP have the ability to activate mouse and human DC, *in vitro* and *in vivo*, with the corresponding IL-12p40/p70, TNF-*α*, and IL-6 production [9, 10]. Therefore, VSSP is a potent adjuvant for DC activation and Th1 differentiation that provide the proper costimulatory context for productive immune responses.

The rate of clinical response observed in this trial was significant although in the absence of randomized data and control arm, it is possible that the regression of high-grade CIN may be due to spontaneous regression [39]. CIGB-228 vaccine treatment met the predetermined criteria of at least a 30% clinical response rate (CR + PR). The observed clinical colposcopic response rate (85.7%), and histological response rate (71.4%) in three months, was more than the estimated spontaneous regression rate of high-grade CIN alone which is estimated to be maximum 10% in 6 months. 

In the current study, immune responses were seen to class I-restricted epitopes of HPV16 E7 protein and regression of CIN as well as diminution of grade of CIN have been associated with the detection of CD8 T cell reactivity to HPV16 E7 protein. There was no clear correlation between HPV16 clearance and clinical response. 

The small number of patients studied and the known spontaneous regression rate of CIN prevents any definitive conclusion as to the utility of the vaccine we tested. Only in futures larger multi-institutional randomized study with a placebo control arm will permit definitive conclusions to be drawn about the effectiveness of CIGB-228 vaccine in the treatment of high-grade CIN. 

The potential of peptides as therapeutic HPV vaccines pass for the difficulties of using peptide antigens for treatment of naturally occurring HPV-induced tumors are the types of HPV causing the tumors (most frequently 16 and 18) and the genetic immunological makeup of the patient (HLA type). However, it is possible to screen patients for the types of HPV present as well as HLA type and it is possible to create a patient-specific vaccine with the appropriate peptide antigens, based on this information. Furthermore, these vaccines are easily produced, are chemically stable, and are devoid of oncogenic potential as well as free of bacterial/viral contaminating substances, hereby avoiding the antigenic competition often seen against viral vector-based vaccines expressing tumor-associated antigens or HIV antigens [40–43] or between simultaneously injected antigens such as in the case with fusion protein or gene products [27, 44, 45].

## 5. Conclusion

In conclusion, our study shows that CIGB-228 vaccination is safe and well tolerated. This vaccine generates promising results based in the levels of immunity and clinical response. It was found to induce robust IFN*γ* T-cell responses to the HPV16 oncoprotein E7 in women with high-grade CIN. CIGB-228 vaccination resulted in clinical activity, as evidenced by the colposcopy, histology and the HPV clearance. In patients with premalignant lesions, immunotherapy with CIGB-228 vaccine is attractive and a trial to confirm therapeutic benefit in such patients has been initiated.

## Figures and Tables

**Table 1:** Results of detection of HPV16 correlated with clinical and immune response.

Patients	Age	HPV 16 before vaccine	HPV 16 prior to LEEP	Initial histology	End histology after LEEP	Clinical status	Immune response
01	43	+	+	CIN II	Koilocytosis	CR	+
02	38	+	+	CIN III	CIN III	SD	+
03	24	+	+	CIN III	CIN II	PR	+
04	25	+	−	CIN II	Koilocytosis	CR	+
05	26	+	−	CIN III	Koilocytosis	CR	+
06	25	+	−	CIN III	Negative	CR	+
07	25	+	+	CIN III	CIN III	SD	+

Note: All patients were positive for HLA-A2.

Initial histology was by cervical biopsy prior to the study. End histology was by LEEP at termination of the study (90 days after the first vaccine administration).

Abbreviations

CIN II-III: cervical intraepithelial neoplasia, grade II or III.

CR: complete response; PR: partial response; SD: stable disease.

**Table 2:** Colposcopic evaluation of patients.

TLA (mm^2^)	Patients						
	01	02	03	04	05	06	07
Initial	244.93	493.68	608.98	638.98	481.71	30.8	291.96
1 month	0 (100%)	462.46 (6.32%)	486.72 (20.07%)	0 (100%)	75.70	0 (100%)	182.10 (37.62%)
2 months	0 (100%)	441.12 (10.64%)	381.8 (37.3%)	0 (100%)	48.15	0 (100%)	149.78 (48.69%)
3 months	0 (100%)	403.2 (18.32%)	286.61 (52.93%)	0 (100%)	0 (100%)	0 (100%)	104.73 (64.12%)

**Response status**	**CR**	**SD**	**PR**	**CR**	**CR**	**CR**	**PR**

Abbreviations

TLA: total lesion area.

TLA: total lesion area.

Complete response (CR) was considered as disappearance of all initial TLA; (PR) partial response if at least a 30% decrease of the initial TLA; progressive disease (PD) meant at least a 20% increase of the initial TLA and as stable disease (SD) if a reduction or an increase of the TLA compared to the initial value that is not enough for classification of the outcome as partial response or progressive disease.

%decrease = TLA_initial_ − TLA_final_/TLA_initial_ × 100.

**Table 3:** Adverse events of patients after each vaccination.

	1st vaccination					2nd vaccination					3rd vaccination					4th vaccination					
Patients	p	a	r	s	sys	p	a	r	s	sys	p	a	r	s	sys	p	a	r	s	sys	
01	x					x					x					x					
02	x	x	x	x			x				x	x				x	x				
03	x					x	x				x						x			1,2,3	
04	x					x	x				x				4	x	x				
05	x										x					x					
06	x	x				x	x				x	x				x	x				
07	x	x			2	x	x				x	x				x	x				

Abbreviations

p, pain at vaccination site.

a, ardor at vaccination site.

r, redness at vaccination site.

s, swelling at vaccination site.

sys, systemic responses. 1-fever; 2-tremors; 3-Lower abdomen pain; 4-cramps.

**Table 4:** IFN*γ* ELISPOT analysis of PBMC before and after vaccinations of patients.

		Patients						
		01	02	03	04	05	06	07
Prevaccination	**86–93**	315	100	70	180	100	160	90
	**82–90**	265	30	80	145	90	90	50
	**11–20**	300	15	80	120	50	75	50
	**M1**	85	85	60	75	50	85	60

After one vaccination	**86–93**	425	145	**190**	190	110	175	110
	**82–90**	260	45	150	150	100	115	60
	**11–20**	315	25	130	130	75	110	60
	**M1**	90	90	75	75	45	90	55

After two vaccinations	**86–93**	540	**305**	125	**530**	**320**	**460**	**220**
	**82–90**	435	**185**	85	**360**	**220**	**420**	**100**
	**11–20**	335	**115**	115	**315**	**230**	**425**	**105**
	**M1**	80	80	55	80	50	80	55

After three vaccinations	**86–93**	**670**	**285**	**190**	**425**	**215**	**510**	**245**
	**82–90**	**710**	**160**	150	**320**	**190**	**445**	**160**
	**11–20**	595	**100**	180	**290**	**155**	**480**	**140**
	**M1**	80	80	55	60	55	80	55

After four vaccinations	**86–93**	**655**	195	**275**	**450**	190	**450**	**200**
	**82–90**	**725**	**140**	**160**	**335**	**180**	**375**	**110**
	**11–20**	550	**105**	**165**	**300**	**130**	**410**	85
	**M1**	75	75	60	80	50	75	60

Note: The PBMCs were tested against three peptide of HPV16 E7 (86–93, 82–90 and 11–20). M1 was taken as positive control. In bold, the positive responses (definition is described in Section 2) are depicted as number of specific spots per 10^6^ PBMCs.
